# *Plasmodium *diversity in non-malaria individuals from the Bioko Island in Equatorial Guinea (West Central-Africa)

**DOI:** 10.1186/1476-072X-5-27

**Published:** 2006-06-19

**Authors:** Ana Guerra-Neira, José M Rubio, Jesús Roche Royo, Jorge Cano Ortega, Antonio Sarrión Auñón, Pedro Berzosa Diaz, Agustín Benito LLanes

**Affiliations:** 1 Centro de Referencia para el Control de Endemias, Centro Nacional de Medicina Tropical. Instituto de Salud Carlos III, Malabo, Bioko, Equatorial Guinea; 2 Servicio de Medicina Tropical. Centro Nacional de Medicina Tropical, Instituto de Salud Carlos III, Sinesio Delgado 6, Pabellón 13, 28220, Madrid, Spain

## Abstract

**Background:**

In this paper we analyse the *Plasmodium sp*. prevalence in three villages with different isolation status on the island of Bioko (Equatorial Guinea) where malaria is a hyper-endemic disease. We also describe the genetic diversity of *P. falciparum*, using several plasmodia proteins as markers which show a high degree of polymorphism (MSP-1 and MSP-2). The results obtained from three different populations are compared in order to establish the impact of human movements and interventions.

**Methods:**

*Plasmodium sp*. were analysed in three villages on Bioko Island (Equatorial Guinea), one of which (Southern) is isolated by geographical barriers. The semi-nested multiplex polymerase chain reaction (PCR) technique was used to determine the prevalence of the four human plasmodia species. The genotyping and frequency of *P. falciparum *populations were determined by PCR assay target polymorphism regions of the merozoite surface proteins 1 and 2 genes (MSP-1 and MSP-2).

**Results:**

The data obtained show that there are no differences in plasmodia population flow between the Northwest and Eastern regions as regards the prevalence of the different *Plasmodium *species. The Southern population, on the other hand, shows a minor presence of *P. malariae *and a higher prevalence of *P. ovale*, suggesting some kind of transmission isolated from the other two. The *P. falciparum *genotyping in the different regions points to a considerable allelic diversity in the parasite population on Bioko Island, although this is somewhat higher in the Southern region than the others. There was a correlation between parasitaemia levels and the age of the individual with the multiplicity of infection (MOI).

**Conclusion:**

Results could be explained by the selection of particular MSP alleles. This would tend to limit diversity in the parasite population and leading up to the extinction of rare alleles. On the other hand, the parasite population in the isolated village has less outside influence and the diversity of *P. falciparum *is maintained higher. The knowledge of parasite populations and their relationships is necessary to study their implications for control intervention.

## Background

Equatorial Guinea is composed of three highly diverse and disparate territories and its total surface area is 28,068 km^2^. It is divided into an insular and a continental region. The island of Bioko represents the main part of the insular region. Bioko Island is part of a volcanic chain formed in the middle and late Tertiary era that extends diagonally across the Gulf of Guinea from the British island of St. Helena (Napoleon's final exile) in the South Atlantic northeast toward Lake Chad, at the northern tip of Cameroon. The more prominent features of this chain include the islands of Annobon, Sao Tome, Principe, and Bioko. Bioko is the largest (2017 km2), highest, and the island nearest to the mainland. The chain also includes Mt. Cameroon, a still active volcano on the mainland itself, which at 4100 m is the highest mountain in West Africa. Bioko Island is roughly rectangular in shape, measuring 69 km from north to south, and 32 km from east to west. It has three major peaks: Pico Basile (3011 m) in the north which serves as a backdrop to the capital city of Malabo; Biao (2009 m) in the southeast, with its picturesque Caldera lake; and the Gran Caldera Volcanica de Luba (2261 m) in the southwest with its steep walls and almost inaccessible interior.

Malaria occupies a major place among the endemic tropical diseases. Over half of the world's population, in approximately one hundred countries, is exposed to malaria. In Africa, between 1,5 and 2,7 million people die of malaria every year [1], and the mayority of deaths take place in children under five years.

Malaria is endemic in Equatorial Guinea, a West-Central African country located in the Gulf of Guinea, with stable transmission and little seasonal change [2]. Malaria is caused by *Plasmodium sp*. infection transmitted by the bite of female mosquitoes of the *Anopheles *genus. *Plasmodium *infection does not correspond directly with malaria disease, and some factors, such as human immunity or specific plasmodia genotypes, may keep the infection level under control. These factors are highly influential in areas of high endemicity where the young children suffer malaria episodes, being these episodes more severe than adults.

Four species traditionally infect humans, *P. falciparum*, *P. malariae*, *P. ovale *and *P. vivax*, but *P. falciparum *is the species that causes more severe illness and it is usually the cause of death. *Plasmodium falciparum *exhibits considerable genomic plasticity that ranges from allelic variation of single genes to chromosomes polymorphism. Several plasmodia proteins, most of them studied for their possible use in vaccine development, show a high degree of polymorphism (Multigene family Pf60, RESA, MSP-1, MSP-2, TRAP, GLURP, CSP, HRP1, ....).

The aims of the study are to analyse the *Plasmodium sp*. prevalence, to describe the genetic diversity of *P. falciparum*, using some of these polymorphic proteins as markers, and to compare the results obtained from three different geographical areas with populations with different grade of isolation and therefore of influence of human movements and interventions.

This might help to understand the genetic structure of the parasite populations in different areas, its relationships with the human population and its evolution in the symptomatic/asymptomatic course of the disease.

## Results

In the assay, the PCR diagnosis showed several differences in relation with microscopy, most of them due to higher sensitivity (table 1). In a single sample, PCR and microscopy identified a different malaria parasite; a *P. falciparum *infection by PCR was identified as *P. ovale *infection by microscopy. PCR data showed a reduction in the number of negative samples together with an increase in the number of mixed infections detected. Nine of the 16 mixed infections detected only by PCR occurred in samples with low parasitaemia for the single species detected by microscopy (<100 parasites/μl), while the remainder occurred in samples with parasitaemia higher than 10,000 parasites/μl (4 samples) or when *P. ovale *was uncharacterised by microscopy (3 samples).

**Table 1:** Comparative study between the SnM-PCR (PCR) and the thin-thick blood smears (Mic).

		**F**	**M**	**O**	**FM**	**FO**	**FMV**	**NEG**
Sacriba	Mic	15	7	1	3			10
	PCR	17	4		6		1	8

Bareso	Mic	14	2					19
	PCR	18	4		4			9

Ureka	Mic	39	3	1	1			37
	PCR	41	2	1	6	3		28

F = *Plasmodium falciparum*; M = *P. malariae*; O = *P. ovale*; FM = *P. falciparum *+ *P. malariae*; FO = *P. falciparum *+ *P. ovale*; FMV = *P. falciparum *+ *P. malariae *+ *P. vivax*; NEG = Negative sample

Results of the parasite prevalence are summarised in table 1. The parasite prevalence rates by microscopy in the three villages were: 72% in Sacriba, 47% in Bareso and 54% in Ureka, while by PCR the prevalence rates were 77%, 74% and 65%, respectively. PCR analysis proved, as expected, to be more sensitive than microscopy, and these results were used to calculate prevalence values. The predominant species was *P. falciparum*, which was present in 67% of the samples in Sacriba, in 63% in Bareso and in 62% in Ureka. The second species was *P. malariae*, which was present in 31% of the samples in Sacriba, 23% in Bareso and 10% in Ureka, showing high variation between regions, although not statistically significant (p > 0.05). *P. ovale *was present only in Ureka (5%), being absent in the other two villages. *P. vivax *appeared in a single sample, a mixed infection in an individual from Sacriba (table 2).

**Table 2:** Prevalence of *Plasmodium sp*. and individualised by species in each village. Percentage of samples with parasitaemia level under 100 p/μl. Percentage of typed *P. falciparum *positives samples, number of alleles found for MSP-1 and MSP-2 and MOI by populations.

	Sacriba	Bareso	Ureka
Prevalence range	70–82%	43–70%	48–60%
Prevalence January 1998			
Microscopy	72%	47%	54%
PCR			
*Plasmodium sp*.	77%	74%	65%
*P.falciparum*	67%	63%	62%
*P.malariae*	31%	23%	10%
*P.ovale*	0%	0%	5%
*P.vivax*	3%	0%	0%
Negatives	22%	26%	35%

Samples Micro. <100 p/μl	31%	44%	43%
Samples PCR. <100 p/μl	36%	65%	53%

Typed samples	79%	55%	66%
			
MSP-1 alleles	10	11	12
MSP-2 alleles	3	4	8
			
MOI	1–5	1–4	1–4

The parasitaemia ranged from 40 to 63,380 parasites/μl of blood, but only four samples had parasitaemia over 1000 parasites/μl. In Sacriba eight out of 26 (31%) positive samples showed fewer than 100 parasites/μl; in Bareso this figure was seven out of 16 (44%) and in Ureka 19 out of 44 (43%). Moreover, if the infections detected only by PCR were included as low parasitaemic infections, then the percentage of samples infected with parasitaemia lower than 100 parasites/μl increased to 36% in Sacriba, 65% in Bareso and 53% in Ureka (table 2).

The level of *P. falciparum *parasitaemia is very important in order to identify the different allele populations present in the sample; with low parasitaemia levels, many samples could not be characterised. Proportion of population with low parasitaemia could affect the prevalence and several alleles in the populations might be underestimated.

In Sacriba, 19 (79%) out of 24 *P. falciparum *positive samples (including mixed infections) could be typed for MSP-1 and/or MSP-2, including two *P. falciparum *mixed infections undetected by microscopy. In Bareso, 12 (55%) out of 22 *P. falciparum *positive samples were typed. Two samples with parasitaemia of 1680 and 1240 parasites/μl were surprisingly negative by PCR typing. In this village none of the *P. falciparum *positive samples by PCR and negative by microscopy showed typing amplification. In Ureka, 33 (66%) out of 50 *P. falciparum *positive samples were typed for MSP-1 and/or MSP-2. Only one out of twelve positive samples by PCR and negative by microscopy showed PCR typing amplification (table 2).

There were 16 alleles identified for MSP-1 (one for the RO33 family, nine for the K1 family and six for the MAD20 family) and eight for MSP-2. MSP-2 showed more diversity in Ureka, where the 8 possible alleles were present in the population, while only 3 and 4 alleles were present in Sacriba and Bareso, respectively (p < 0.05). These differences were not marked in MSP-1, where 10, 11 and 12 alleles were present in Sacriba, Bareso and Ureka, respectively (table 2). The allelic frequency in each village was clearly different, with several autochthonous alleles (table 3), especially in Ureka (e.g. K_150_, Mp_400_).

**Table 3:** Alleles identified in each population, numbers (N) and frequencies (*fx*). The different alleles are represented by the first letter of the corresponding family for MSP-1 (R, K and M) and Mp for MSP-2, accompanied by the size of the PCR product.

	Sacriba		Bareso		Ureka	
	N	*fx*	N	*Fx*	N	*fx*

MSP-1						

R_150_	9	0.23	5	0.19	14	0.18

K_100_	5	0.13	-	0.0	5	0.06
K_125_	-	0.0	2	0.12	-	0.0
K_140_	-	0.0	1	0.06	-	0.0
K_150_	-	0.0	-	0.0	19	0.24
K_160_	-	0.0	1	0.06	5	0.06
K_175_	-	0.0	3	0.19	3	0.04
K_200_	9	0.23	1	0.06	15	0.19
K_225_	3	0.08	3	0.19	1	0.01
K_250_	3	0.08	2	0.12	-	0.0

M_175_	-	0.0	-	0.0	2	0.02
M_200_	2	0.05	4	0.25	2	0.02
M_225_	4	0.10	-	0.0	7	0.09
M_250_	2	0.05	2	0.16	5	0.06
M_275_	1	0.03	2	0.16	1	0.01
M_300_	1	0.03	-	0.0	-	0.0

MSP-2						

Mp_300_	-	0.0	1	0.12	1	0.02
Mp_400_	-	0.0	-	0.0	9	0.21
Mp_450_	5	0.50	-	0.0	5	0.12
Mp_500_	4	0.40	4	0.50	10	0.23
Mp_550_	1	0.10	2	0.25	10	0.23
Mp_600_	-	0.0	1	0.12	6	0.13
Mp_650_	-	0.0	-	0.0	1	0.02
Mp_700_	-	0.0	-	0.0	1	0.02

The minimal number of different *P. falciparum *infections found in a single sample (Multiplicity of infection: MOI) corresponds to the larger number of alleles for MSP-1 or MSP-2 by sample. In our study the MOI ranged from 1 to 4 in Bareso and Ureka, and from 1 to 5 in Sacriba. The MOI data showed that there was a direct correlation (p < 0.05) between the number of *P. falciparum *populations and parasitaemia. Fifteen samples (34%) with a parasitaemia higher than 100 parasites/μl showed MOI equal to or higher than three while for parasitaemia under 100 parasites/μl only 4 samples (9%) had MOI equal to 3 or 4 (table 4).

**Table 4:** Relation between MOI and parasites/μl by sample in the three populations. MOI = Multinfection index. Pobl. Villages. S: Sacriba; B: Bareso; U: Ureka.

	MOI number																	
	0			1			2			3			4			5		

Villages	S	B	U	S	B	U	S	B	U	S	B	U	S	B	U	S	B	U

Parasites/μl																		
<100	4	8	15	2	4	5	1	3	-	1	-	1	-	-	2	-	-	-
<1000	1	2	-	5	1	4	5	1	7	1	1	6	1	-	3	1	-	-
>1000	-	-	-	1	-	-	-	1	-	-	1	-	1	-	-	-	-	-

Moreover, there was a minor inverted correlation between MOI and age. Only 36% of the children under 5 years old had MOI of 0 or 1, while in children between 5 to 10 years of age this value increased to 60%, and in children over 10 years of age to 70% (p < 0.05) (table 5).

**Table 5:** Relation between MOI and age in the three populations. MOI = Multinfection index. Villages: S: Sacriba; B: Bareso; U: Ureka

	MOI number																	
	0			1			2			3			4			5		

Villages	S	B	U	S	B	U	S	B	U	S	B	U	S	B	U	S	B	U

Age																		
< 5	1	1	2	3	1	2	4	2	2	3	1	2	2	-	2	1	-	-
= 5–10	1	9	2	2	3	2	2	3	2	2	1	2	-	-	1	-	-	-
> 10	3	-	9	3	1	5	-	-	3	1	-	3	-	-	2	-	-	-

## Discussion

Plasmodia characterisation by PCR increases the specificity and sensitivity of the traditional thin-thick smears [3,4]. All discrepancies between PCR and microscopy diagnoses may be explained in terms of the higher sensitivity of the PCR. In one case there was a possible misdiagnosis, a *P. falciparum *infection by PCR was characterised as *P. ovale *by microscopy, due probably to a very low parasitaemia (40 parasites/μl). These improvements in sensitivity allow a better approach to the real parasite prevalence in the population, particularly when the target population in this study consists of non-malaria individuals. Therefore, most of the individuals studied had null o very low parasitaemia.

In the study, the prevalence is shown to be around 75% in Sacriba and Bareso (Northwest and East regions), while in Ureka (South) it decreases to 65%, although this difference is not statistically significant.

In Sacriba and Bareso, the prevalence data for each *Plasmodium *species are similar, and also similar to previous reports carried out in the North part of the Island [3], with some variations in the less represented species. In any case, despite the absence of *P. ovale *in both populations, there is a occasional transmission of *P. vivax *in Sacriba, maintained probably by a semi-immune mulatto population with the absence of a specific membrane receptor (Duffy blood group) that the parasite needs for the invasion of red cells [3]. Duffy-blood-group-negative human erythrocytes, FyFy, are resistant to invasion by *Plasmodium vivax*. The FyFy genotype is found predominantly in African and American Blacks, who are the only groups resistant to infection by P.vivax. On the other hand, in Ureka, there is a trend to a lower prevalence of *P. malariae *(15 to 20% less) in comparison to the other two villages and to previous reports, although it was not statistically significant. This, together with the higher parasite prevalence, 77% and 75% in Sacriba and Bareso respectively, as opposed to 65% in Ureka, suggests some kind of population isolation breaking the parasite flow between the northern and south parts of the Island. These data argue in favour of the results of Roche et al. [2], who detected a higher presence of *P. malariae*, in the continental region (Fang ethnic population) than on the Bioko Island (mainly Bubi ethnic population). At the same time, they observed a higher presence of *P. malariae *in the Fang population of the Island, suggesting that these differences were due to increased migration from the continental region. This migration is traditionally to the northern part of the Island, while there is a lower level of migration to the south (mainly Bubi ethnic population), which is isolated by geographical barriers. This could be the explanation for the lower presence of *P. malariae *infection and the lower prevalence of malaria parasites, suggesting that the differences found between villages could be partly a result of human movements.

*Plasmodium falciparum *polymorphism for the MSP-1 and MSP-2 loci were analysed in the three villages. MSP-1 and MSP-2 alleles were defined according to length of the fragments. Several samples with low parasitaemia could not be typed or were under-typed, showing a direct correlation between parasitaemia and typing that implies that several alleles in the *P. falciparum *population might be underestimated. Consequently, the direct correlation between the MOI number and parasitaemia could be an effect of the lower sensitivity of PCR genotyping in comparison to the SnM-PCR used for the diagnosis [5,6].

Otherwise, the inverse correlation between the MOI number and age might reflect acquired antiparasite immunity. This immunity is present in children over 5 years of age, earlier than reported by other authors. Smith et al. [6] described a multiplicity peak at the level of 2–7 year-old children, with MOI decreasing after that age; Konate et al. [7] described the MOI peak in children between 5 to 9 years old, with MOI decreasing very slowly afterwards. Our data show that in children over 5 years of age on Bioko Island, the MOI number decreases. Our possible explanation is that the transmission rate is so high in Equatorial Guinea (entomological inoculation rates-EIR varied between 242–281 bites/person/night for *Anopheles gambiae s.s*. and 317–787 bites/person/night for *An. funestus *[8]) that children become infected earlier with different *Plasmodium *parasites that in other endemic countries, where children acquire immunity later.

The anopheline density on Bioko Island is lower than in most West-African-tropical countries and on islands such as Sao Tomé and Principe [9]. Nevertheless, the low mosquito density is compensated by one of the highest infective rate in West-Central Africa [8,10]. These high infective rate increases the number of possible sexual recombinations into the mosquito, which could booster the number of alleles present in the populations, as occurs on Bioko Island in comparison to other areas with similar transmission rates [7,11]. The chance of there being a high allele recombination rate in the mosquitoes could result in higher number of alleles being present in the population and, at the same time, would decrease allelic population homogeneity between villages, as occurs in our study area.

MSP-2 showed a significantly higher diversity in Ureka than in the other two northern villages and we also found there autochthonous alleles such as K_150 _and Mp_400_. This data could be explain at least in part by the effects of the malaria control activities that could have resulted in the selection of particular MSP alleles, maybe in some cases associated with drug resistance. This would tend to limit diversity in the parasite population and move towards the extinction of rare alleles. On the other hand, the human movements in the isolated village are fewer, the control activities are rare and the number of drugs available for treatment is lower. Therefore, its parasite population has less outside influence and the diversity of *P. falciparum *is maintained higher.

## Conclusion

This data could be explained by the selection of particular MSP alleles. This would tend to limit diversity in the parasite population and leading up to the extinction of rare alleles. On the other hand, the parasite population in the isolated village has less outside influence and the diversity of *P. falciparum *is maintained higher. The knowledge of parasite populations and their relationships is necessary to study their implications for control intervention.

Future studies using molecular markers and an extensive field trial will help to understand – for cut off regions such as Bioko – the relation between prevalence rate, loci variability and vector behaviour and genetic variability in relation to intervention measures for control, such as the designing of future vaccines.

The accomplishment of a study with more number of samples will allow obtaining comparisons of statistical signification necessary to corroborate these preliminary data.

## Methods

### Area of study

Bioko Island is an 800 square mile island located 20 miles (32 km) off the coast of Cameroon in west Central Africa. Bioko, formerly Fernando Po, is a part of the African country of Equatorial Guinea, known as Spanish Guinea before its independence from Spain in 1968. Equatorial Guinea also includes Rio Muni, a mainland rectangle of land between Cameroon and Gabon, and the island of Annobon, another one of the Gulf of Guinea islands. Equatorial Guinea's capital city of Malabo (pop. 60,000) is located on Bioko's northern tip.

**Figure 1:**
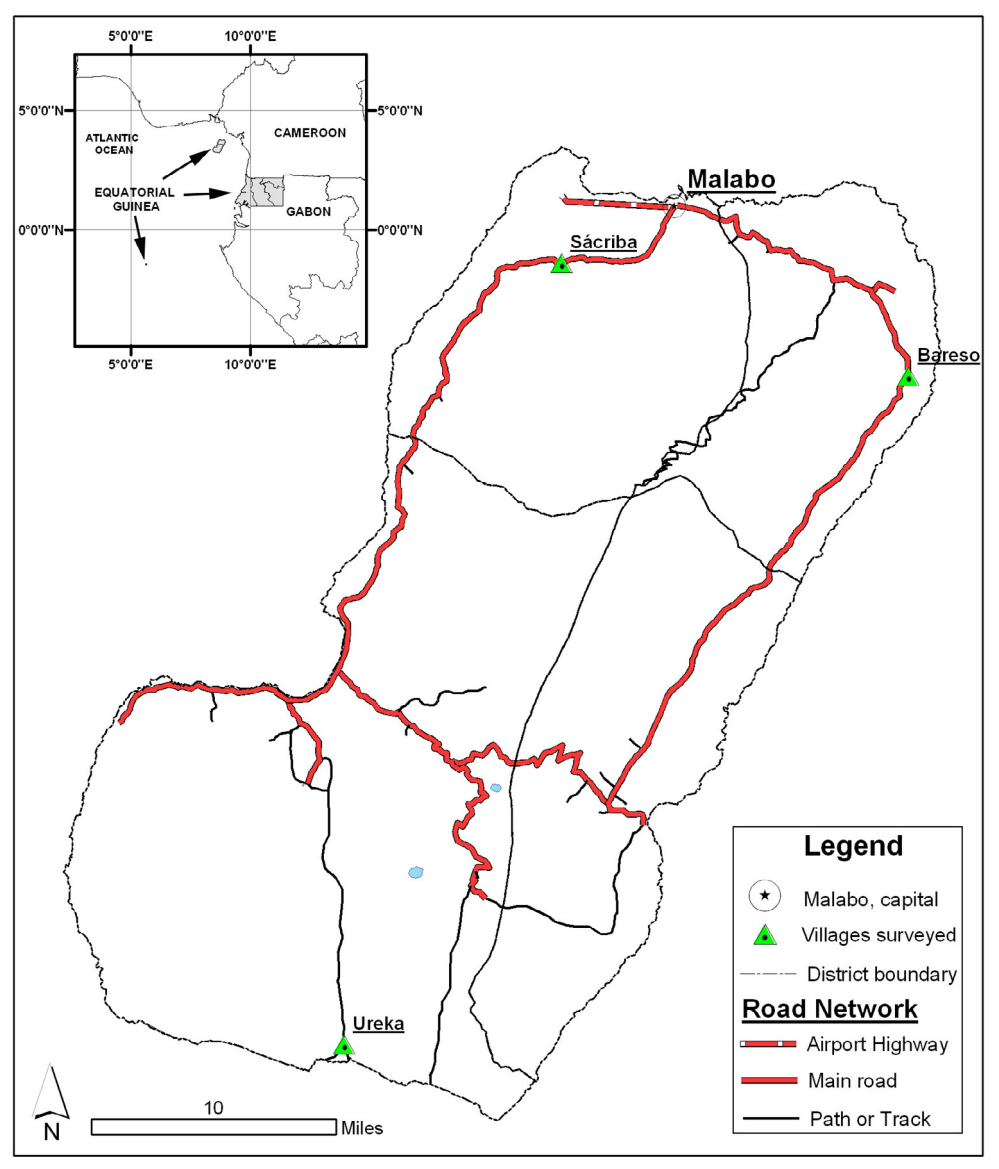
Map of Equatorial Guinea and geographical situation of Bioko Island. Location of the sampled villages on Bioko Island.

Bioko Island is one of the main regions of the Republic of Equatorial Guinea, a West-Central African country located in the Gulf of Guinea (figure 1). Bioko, with 70,000 inhabitants and a surface area of 2,017 km^2^, lies 30 km to the west of the coast of Cameroon. Malabo is the main town of the island and the capital of the nation. On Bioko, there are only two seasons: rainy (from May to October) and dry (November to April).

Three populations, one for each of the three health areas into which the Bioko Island is subdivided, were selected for the study: Bareso 3° 38'60N; 8° 55'0E (East), Sacriba 3°42'0N ; 8° 43'0E (Northwest) and Ureka 3° 16'0N; 8° 31'60E (South). The size of all of them is between 100 and 300 inhabitants. Bareso and Sacriba are about 15–25 kilometres from Malabo, the capital, and there is a road linking them. Ureka, a village of only 100 people, is an 8 to 10 hour downhill hike from Moca, although most people get there by an 8 hour open boat ride and then a 1 to 2 hour walk along the beach. The ocean water is warm and the waves may reach as high as 10 ft. Getting out of Ureka is as difficult as getting in and it is isolated by geographical barriers, such as a volcano (Gran Caldera de Luba), the tropical forest and the lack of roads (figure 1).

### Sample collection

The survey was carried out in January 1998 in the three villages where transmission is perennial. Samples were collected actively from all asymptomatic residents under 10 years old. Thick and thin films were stained with 10% giemsa and examined by two independent technicians. Finger prick samples on filter paper (36 from Sacriba, 35 from Bareso and 81 from Ureka) were also obtained. Each filter paper specimen was stored in a plastic bag at room temperature. Ethical considerations were accepted by the Ethical Committee for both Institutions and agreement to be included in the survey was given by the parents.

### DNA isolation and species identification by PCR

DNA extraction was carried out using the Chelex method [12] with minor modifications [3]. Detection of *Plasmodium sp*. infection and identification of malaria species was done simultaneously using a semi-nested multiplex amplification [3].

### Analysis of multiple Plasmodium falciparum infections

MSP-1 (K1, MAD20 and RO33 allelic families) and MSP-2 analyses were performed according to Robert et al. [13], except that no hybridisation was carried out to differentiate the MSP-2 allelic families (FC27 and 3D7). We preferred not to do a hybridisation as it would have increased the sensitivity of MSP-2 detection and the comparison with MSP-1 alleles would not be under the same conditions. The alleles were named by the first letter of the corresponding family for MSP-1 (R, K and M) and Mp for MSP-2, accompanied by the size of the PCR product.

The size of the amplified fragments was measured by MultiAnalyst Software – Gel Doc 1000 (BioRad®) (SE = ± 20 bp) from 2% agarose gel electrophoresis with ethidium bromide staining.

### Data analysis

Statistical variance analysis and χ^2 ^was performed with Epi Info 6.04 (CDC, Atlanta, USA and WHO, Geneva, Switzerland).

## Abbreviations

PCR: polymerase chain reaction

MOI: multiplicity of infection

RESA: ring-infected erythrocyte surface antigen

MSP-1: merozoite surface protein 1

MSP-2: merozoite surface protein 2

TRAP: Thrombospondin-related adhesive protein

GLURP: glutamate-rich protein

CSP: circumsporozoite surface protein

HRP1: histidine-rich protein-2

## Competing interests

The author(s) declare that they have no competing interests.

## Authors' contributions

AGN and PBD carried out the molecular study and analysed the results; JMR was the molecular biologist that participated on design and analysis of the results; JRR was the coordinator of the Project in Equatorial Guinea and participated designing the study; JCO and ASA co-operated in the epidemiological study an collected the samples on paper filter from the population studied in Equatorial Guinea and coordinated the optical microscopic diagnosis; ABL conceived of the study and participated in its design and coordination. All authors read and approved the final manuscript.
